# Application of Effective Day Degrees in the Assessment of Stable Isotope Patterns in Developing Seahorses under Different Temperatures

**DOI:** 10.3390/ani10091571

**Published:** 2020-09-03

**Authors:** Sonia Valladares, Miquel Planas

**Affiliations:** Department of Ecology and Marine Resources, Instituto de Investigaciones Marinas (CSIC), Eduardo Cabello 6, 36208 Vigo, Spain; svallalago@gmail.com

**Keywords:** seahorse, effective day degrees, temperature, stable isotopes, *Hippocampus*

## Abstract

**Simple Summary:**

Temperature affects fish development, with especially strong influence on juvenile growth rates and metabolism. The present study provides new insights on stable isotopes (δ^13^C and δ^15^N) for the understanding of growth and food assimilation in early developing European long-snouted seahorse *Hippocampus guttulatus* under different temperature levels. The effects of feeding status, ontogeny and temperature regimes on stable isotope patterns were assessed and modelled as function of development. We argue that chronological time is not a convenient developmental scale and we encourage the use of D°_eff_ as temperature-independent developmental index in stable isotopes studies involving temperature comparisons.

**Abstract:**

Relations between nutrient assimilation and growth rate in fishes may vary with abiotic factors such as temperature. The effects of feeding status, ontogeny and temperature regimes (15, 18 and 21 °C) on stable isotope (δ^13^C and δ^15^N) patterns were assessed in juveniles of the seahorse *Hippocampus guttulatus*. The use of effective day degrees (D°_eff_), day degrees (D°) and chronological time (age) were compared as development progress indices. Newborn seahorses were maintained at three temperature levels both deprived of food (5 days) or fed (30 days) on copepods or/and *Artemia*. Isotopic signatures in fed seahorses clearly differed from those in unfed juveniles. Temperature had a significant effect on δ^13^C values in fed juveniles throughout the experimental period. δ^15^N values also varied significantly with age, but not with temperature level. Faster growth and food assimilation in seahorses held at 18 and 21 °C were supported by faster variations in isotopic values. Our findings demonstrate that effective day degrees should be preferred over chronological time as index of developmental progress in temperature fluctuating scenarios or for comparative studios.

## 1. Introduction

The estimation of food intake, digestibility and assimilation patterns provides valuable information for the interpretation of growth and mortality rates of a consumer [1,2]. Indirect techniques used to determine nutrient assimilation in fish (e.g., faeces collection, gut content analysis or individual growth rate measurement) might be difficult to apply, particularly in early life stages, due to size limitation, complexity of sample collection and quantification of food intake [2,3]. A direct method for overcoming these difficulties is the use of stable isotopes, whose values in consumer tissues reflect those of the food incorporated plus a trophic discrimination factor that occurs with nutrient assimilation [4]. For dietary studies, the two most commonly measured stable isotope ratios are ^15^N/^14^N and ^13^C/^12^C; both ratios are usually higher in consumer tissues compared to its diet because the lighter isotope (^14^N and ^12^C) is preferred in metabolic processes [4,5]. Even though high variation has been reported [6], it is usually assumed that trophic discrimination factors (Δδ) are 0–1‰ for δ^13^C [4,7,8] and 3.4‰ [5,9,10] for δ^15^N, depending on tissues/species considered [11].

Carbon and nitrogen stable isotopes (^13^C and ^15^N) have been successfully used as dietary tracers for assessing the food utilization by organisms [12,13,14,15]. Numerous factors such as environmental conditions (e.g., temperature), feeding rates, physiological and nutritional status of the consumer (e.g., stress, starvation) often cause modifications to food assimilation and thus differences in consumer isotope composition [16,17,18,19]. Experimental feeding studies allow the isolation of one or more factors that modulate stable isotope ratios in consumers. In the case of fish larvae, experimental stable isotope studies investigating the effects of environmental conditions on stable isotope incorporation are relevant in identifying environmental preferences of larvae, understanding larval nutrition needs, improving rearing techniques, and interpreting field stable isotope studies.

In lecitotrophic larvae of teleosts, initial isotopic trends would at least partially depend on the presence and quantity of yolk remaining in the yolk-sac at hatching. Conversely, juvenile seahorses are fully developed, active swimmers and hunters, and exclusively dependent on exogenous feeding immediately after male’s pouch release, when yolk is almost exhausted [20]. Suboptimal nourishment or starvation during the first life stages of seahorses would cause the mobilization of endogenous reserves from tissues to support energetic and metabolic demands, resulting in changes for δ^13^C and δ^15^N signals, which would differ from those in fed individuals.

Generally, stable isotope values are fitted according to growth/weight or time-based models [21,22,23]. The use of time-based models is practical when using chronological time (days) but not for fitting and comparing data from different temperature conditions. Temperature affects nearly every aspect of fish development, with strong influence on larval and juvenile growth rates and metabolism [24,25,26,27,28,29]. Effective day degrees (D°_eff_) is a temperature independent index of development progress in poikilotherms [26]. Planas et al. [29] demonstrated for the first time the suitability of D°_eff_ as a temperature-independent index to quantify development and growth in feeding juveniles of a viviparous fish, the seahorse *Hippocampus guttulatus*.

The direct effect of temperature on stable isotopes has been investigated in a few marine fish species [17,19,30,31], but never in syngnathid fishes such as seahorses. The present study was carried out: (1) to test the hypothesis that fish developed at optimal temperature conditions will exhibit maximal growth and nutrient assimilation rates, which would be reflected in the rate of change of consumer isotopic signatures, and (2) to assess the applicability of D°_eff_ as development index in modelling stable isotope patterns. The study was performed in the early life stages of the seahorse *H. guttulatus* by assessing the influence of three temperature levels on changes in carbon (δ^13^C) and nitrogen (δ^15^N) stable isotope values in fed or starved seahorse juveniles. To our knowledge, the present study supports the use of the D°_eff_ approach in the assessment of stable isotope patterns in animals for the first time.

## 2. Materials and Methods

### 2.1. Broodstock

Adult seahorses *Hippocampus guttulatus* Cuvier, 1829 were collected in Galicia (NW Spain) and maintained in ad hoc aquaria [32] at Instituto de Investigaciones Marinas (IIM-CSIC) (Vigo, Spain). Sea water temperature was maintained within an annual temperature regime ranging from 15 °C in winter to 19 °C in summer (±0.5 °C). A natural-like photoperiod regime was applied: 10L: 14D in winter and 16L: 8D in summer. Pumped seawater was filtered (5 µm), UV treated, and 10–15% daily exchanged. Water quality was checked periodically for NO_2_, NO_3_ and NH_4_/NH_3_ content (0 mg L^−1^) using Sera Test Kits. Salinity and pH levels were maintained constant at 38 ± 1 and 8.1 ± 0.1, respectively. Seahorses were fed ad libitum twice daily on a diet consisting of nutritionally-enriched adult *Artemia* (EG, Inve, Cádiz, Spain) supplemented with captured mysidaceans (*Leptomysis* sp. and *Siriella* sp.).

### 2.2. Fed Seahorses

Two batches of seahorse juveniles were released by two males held in captivity for 19 months. Immediately after male’s pouch release, juveniles from each batch were randomly transferred (5 juveniles L^−1^) into twelve 30 L pseudo-Kreisel aquaria (2 aquaria per batch and temperature level) [33]. The rearing system was illuminated by 20 W fluorescent lamps (Power Glo) and submitted to a 16L: 8D photoperiod regime. Water temperature was initially adjusted to 15 °C and subsequently increased for 2 days until reaching the desired experimental temperatures: 15, 18 and 21 °C (±0.5 °C). Total seawater volumes in the rearing system were replaced twice per hour by means of an external inflow (24 L h^−1^) of 20 μm filtered and UV-treated seawater. Aquaria were gently aerated in the upper part of the water column at a continuous flow rate of 700 mL min^−1^.

Seahorse juveniles were fed for 30 days according to an optimized feeding schedule for growth and survival maximization [34]. Three feeding periods were established from male’s pouch release (day 0):

- First feeding (days 0 to 5): Single daily dose of cultivated copepods *Acartia tonsa* and *Tisbe* sp. (1:1; 0.6 copepods mL^−1^).
- Transitional feeding (days 6 to 10): Daily dose of copepods (0.3 copepods mL^−1^) and Great Salt Lake *Artemia nauplii* (1 *Artemia* mL^−1^).
- *Artemia* feeding (days 11 to 30): Three daily doses of *Artemia nauplii* and 24 h enriched *Artemia metanauplii* (1:1; 1 *Artemia* mL^−1^).

Copepods were cultivated in 250–500 L tanks at 26–27 °C and 38 salinity and fed every two days on mixtures of the microalgae *Isochrysis galbana* and *Rhodomonas lens* (10^3^ cells mL^−1^). Only copepods retained by a 125 μm mesh were offered to seahorses. *Artemia* was nutritionally enriched in 5 L buckets (26 °C, 100 *Artemia* mL^−1^). The enrichment diet consisted of a mixture of the microalgae *Isochrysis galbana*, *Phaeodactylum tricornutum* and *Rhodomonas lens* (10^7^ cells mL^−1^). Twice daily, wastes and faeces were siphoned out, and dead seahorses removed and counted.

### 2.3. Unfed Seahorses

Seahorse juveniles were obtained from the batches reported for the feeding experiment and maintained deprived of food until total mortality at an initial density of 2 juveniles L^−1^ (two 30 L pseudo-Kreisel aquaria per batch and temperature level) with a constant water flow rate of 300 mL min^−1^ and moderate aeration. Mortalities were recorded daily throughout the experimental period.

### 2.4. Bioethics

Animal maintenance and manipulation practices were conducted in compliance with all bioethics standards of the Spanish Government (Real Decreto 1201/2005, 10th October 2005) and approved by the Bioethics Committee of IIM-CSIC. Sampled juveniles were anesthetized or euthanized using tricaine methane-sulfonate (MS-222, Sigma-Aldrich, Darmstadt, Germany) at a concentration of 0.1 mg L^−^^1^ or above.

### 2.5. Sampling, Analyses and Data Treatment

At the onset of the experiments, seahorse juveniles were subsampled (n = 10 per batch) to determine initial carbon (δ^13^C) and nitrogen (δ^15^N) isotope values, weight and length. Samples of *Artemia* and copepods were also collected, rinsed with distilled water and kept frozen at −20 °C for further isotope analysis. In the feeding experiment, samples for isotopes, weight and length analysis in juveniles were randomly collected (n = 4 per treatment) at ages of 5, 15 and 30 days from each aquarium before first daily feeding. Starved seahorses were sampled at day 5 (n = 10 per treatment), prior to 50% mortality (5.6–6.7 days, depending on temperature) [29].

Sampled juveniles were anesthetized with tricaine methane-sulfonate MS222 (0.1 g L^−1^), transferred to Petri dishes, photographed and weighed individually on a Sartorius microbalance (±0.01 mg). Standard lengths (SL) were measured according to Lourie et al. [35] (SL = head + trunk + curved tail) from digital photographs using an image processing software (NIS Elements, Nikon Tokyo, Japan).

For isotope analysis, the seahorses were rinsed with distilled water, frozen at −20 °C, freeze dried and homogenized. The analyses were made in bulk seahorses on sub-samples of 1 mg dry weight biomass. High lipid content in samples might cause significant alterations in δ^13^C and, to a lesser extent, δ^15^N values for most species and tissue types, indicating the need to correct for lipid carbon isotope effects [36]. Samples are lipid extracted prior to the analysis when lipid content exceeds 5% weight (C:N > 3.56) [37]. C/N values in our samples indicated that lipid content was higher than 5% in some samples, particularly in prey. We did not perform lipid extraction on the samples. Instead, our own correction factors were applied to seahorse juveniles, copepods and *Artemia*.

δ^13^C and δ^15^N values and elemental composition (total C and N percentage) were analyzed at Servizos de Apoio á Investigación (SAI) of the University of A Coruña (Spain). Samples were measured by continuous flow isotope ratio mass spectrometry using a FlashEA1112 elemental analyser (Thermo Finnigan, Monza, Italy) coupled to a Delta Plus mass spectrometer (FinniganMat, Bremen, Germany) through a Conflo II interface. Carbon and nitrogen stable isotope abundance was expressed as permil (‰) relative to VPDB (Vienna Pee Dee Belemnite) and Atmospheric Air, according to the following equation:

δX = (R_sample_/R_reference_) − 1, (1)

where X is ^13^C or ^15^N and R is the corresponding ratio of ^13^C/^12^C or ^15^N/^14^N. As part of an analytical batch run, a set of international reference materials for δ^15^N values (IAEA-N-1, IAEA-N-2, IAEA-NO-3) and δ^13^C values (NBS 22, IAEA-CH-6, USGS24) were analyzed. The precision (standard deviation) for the analysis of δ^13^C and δ^15^N of the laboratory standard (acetanilide) was ±0.15‰ (1-sigma, n = 10). Standards were run every 10 biological samples.

Changes in δ^13^C and δ^15^N were studied by applying two different developmental index: chronological time (days) and effective day degrees (D°_eff_). Effective day-degrees (D°_eff_) is a temperature independent index of developmental progress based on a species-specific threshold temperature (T_o_) at which development is theoretically arrested [26]. D°_eff_ was calculated as:

D°_eff_ = Δt T_eff_ = Δt (T − T_o_), (2)

where Δt is developmental time in days, T_eff_ is the biologically effective temperature (T_eff_ = T − T_o_) and T_o_ the threshold temperature for *H. guttulatus* juveniles (13.1 ± 0.9 °C) [29].

Values are provided as mean ± standard deviation. A Shapiro-Wilk test was used to test for normality of variables. Analysis of variance (ANOVA Univariate General Linear Model) was applied to estimate the effects of temperature on survival, growth parameters and isotope data. When ANOVA assumptions were not met (Levene’s test of homogeneity and Bartlett’s test of homoscedasticity), non-parametric Kruskal-Wallis tests were applied instead. When significant differences were found at an alpha value of 0.05, Tukey’s HSD post-hoc test was applied to determine significance of pairwise differences. Statistical analyses and model-fitting were performed with Statistica 8.0 (StatSoft, Tulsa, OK, USA) software package.

## 3. Results

### 3.1. Growth, Survival and Condition of Juveniles

Unfed juveniles showed weight loss at all tested temperatures but slightly increased in length (about 1 mm until day 5) (Table 1). Juvenile survivals at day 5 were 88, 94 and 89% at 15, 18 and 21 °C, whereas full mortalities were recorded at days 9, 8 and 7, respectively. In fed seahorses, the highest final survival occurred at 18 °C (86 ± 0.4%), which was significantly higher than at 15 °C (21 ± 2%) and 21 °C (81 ± 0.2%) (Kruskal-Wallis test, *p* < 0.05). First mortalities started at day 4 in 15 °C treatment and beyond day 6 at 18 and 21 °C. Final dry weights (day 30) at 15, 18 and 21 °C were 1.53 ± 0.39, 7.57 ± 7.28 and 12.79 ± 10.20 mg, respectively (Table 1). Despite clear differences among treatments, final weights did not differ significantly with temperature due to the large standard deviations of means at 18 and 21 °C (F_(2,5)_ = 1.21, *p* = 0.41). C:N values were rather constant (<2.94) and did not differ significantly across temperature levels (F_(2,5)_ = 1.02, *p* = 0.39) (Table 1).

### 3.2. Isotopic Patterns with Ontogeny and Feeding Conditions

**Figure 1:**
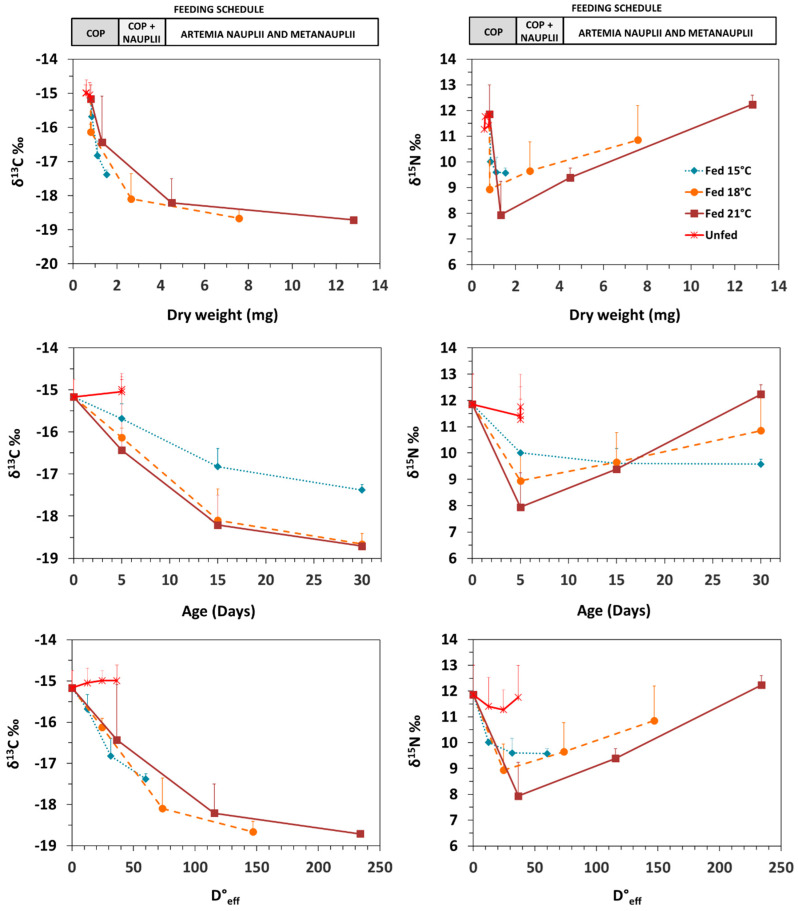
Changes in δ^13^C and δ^15^N values (‰) in seahorse *Hippocampus guttulatus* juveniles grown at 15, 18 and 21 °C under feeding (gray line) and food deprivation (unfed; black line) conditions. Data are provided as means (two batches per temperature level) for dry weight (mg; upper) chronological time (days; middle) and effective day-degrees (D°_eff_; below). Prey: copepods (cop) and *Artemia* (nauplii and metanauplii).

The average isotopic values for copepods, *Artemia* nauplii and metanauplii were −18.62, −20.27 and −19.15‰ for δ^13^C and−1.47, 12.30 and 9.35‰ for δ^15^N, respectively. Average δ^13^C and δ^15^N values in newborn seahorses were −15.17 ± 0.42‰ (n = 10) and 11.86 ± 1.15‰ (n = 10), respectively (Figure 1).

**Figure 2:**
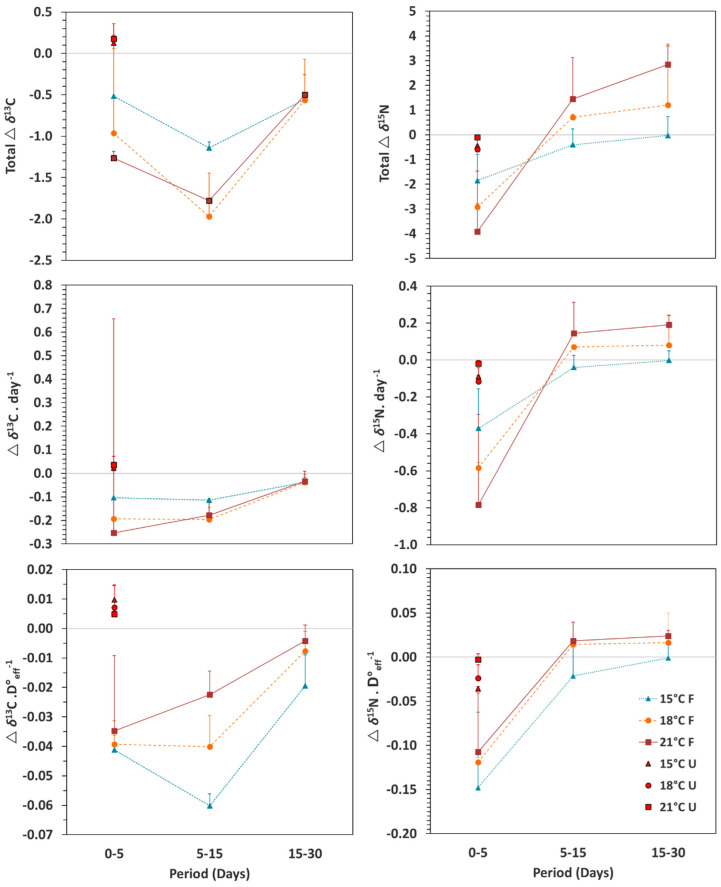
Changes in δ^13^C and δ^15^N (‰) within feeding periods (days 0–5, 5–15 and 15–30) in seahorse *Hippocampus guttulatus* juveniles maintained at 15, 18 and 21 °C under feeding (F; solid symbols) or food deprivation, unfed (U; open symbols).

Non-significant isotopic changes occurred in unfed seahorses from days 0 to 5 (Figure 1; Figure 2). At 15, 18 and 21 °C, those changes corresponded to total δ^13^C increase of 0.45, 0.58 and 0.10‰ and δ^15^N decrease of 0.12, 0.18 and 0.18‰, respectively.

In fed seahorses, a progressive asymptotical decrease in δ^13^C values occurred from first feeding until the end of the experiment (progressive approach to diet values), whereas δ^15^N decreased initially and afterwards increased sharply during the *Artemia* feeding period. As shown in Figure 1, due to differences in temperature levels and in the resulting differences in developmental progress of juveniles across temperatures, chronological time (age) did not provide an adequate reference scale for development. On the contrary, weight and effective-day degrees (D°_eff_) performed similarly. In the first feeding stage (copepods), isotopic decreases were recorded at 15, 18 and 21 °C, accounting for 0.52, 0.96 and 1.27‰ in δ^13^C and 1.85, 2.92 and 3.92‰ in δ^15^N (Figure 2). Daily decrease rates in δ^13^C and δ^15^N were directly correlated with temperature level, ranging from 0.10 to 0.25‰ day^−1^ and from 0.37 to 0.78‰ day^−1^, respectively (Figure 2). Considering D°_eff_ as developmental scale, decrease rates were similar and not related to temperature level, (0.03–0.04‰ D°_eff_^−1^ in δ^13^C; 0.10–0.13‰ D°_efff_^−1^ in δ^15^N) (Figure 2).

The transition from copepods to *Artemia* feeding was characterised by a drop in δ^13^C values at all temperature levels, a small decrease in δ^15^N values at 15 °C, and an increase in δ^15^N values at 18 and 21 °C (Figure 1; Figure 2). At day 15, δ^15^N and δ^13^C values across treatments were similar (F_(2,5)_ = 0.07, *p* = 0.94 and F_(2,5)_ = 2.86, *p* = 0.20, respectively); daily decrease in δ^13^C at 15 °C (0.10‰ day^−1^) was lower than at 18 °C (0.19‰ day^−1^) and 21 °C (0.25‰ day^−1^) (Figure 2). For δ^15^N, daily changes accounted for −0.04, 0.07 and 0.15‰ day^−1^ at 15, 18 and 21 °C, respectively (Figure 2). Regarding isotopic variation relative to D°_efff_, changes were not significantly different among treatments in δ^13^C (−0.06, −0.04 and −0.02‰ D°_efff_^−1^ at 15, 18 and 21 °C, respectively; F_(2,5)_ = 10.98, *p* = 0.04), except for δ^13^C at 15 and 21 °C (*p* = 0.04), nor in δ^15^N (−0.02, 0.02 and 0.02‰ D°_eff_^−1^ at 15, 18 and 21 °C, respectively; F_(2,5)_ = 1.76, *p* = 0.31).

The period of feeding on *Artemia* nauplii and metanauplii, comprising days 11 to 30, led to a progressive decrease in δ^13^C values (final values of −17.38, −18.66 and −18.71‰ at 15, 18 and 21 °C, respectively) and an increase in δ^15^N values (final values of 9.58, 10.86 and 12.24‰ at 15, 18 and 21 °C, respectively) (Figure 1; Figure 2). At 15, 18 and 21 °C, those changes corresponded to −0.02, −0.01 and −0.00‰ D°_eff_^−1^ for δ^13^C (F_(2,5)_ = 2.08, *p* = 0.27), and −0.00, 0.02 and 0.02‰ D°_eff_^−1^ for δ^15^N (F_(2,5)_ = 0.52, *p* = 0.64), respectively.

## 4. Discussion

Temperature independence of effective day degrees (D°_eff_) as an index of development progress in poikilotherms was firstly demonstrated by Weltzien et al. [26]. In addition, the suitability of that index to quantify development and growth in feeding larvae/juveniles of viviparous fishes, particularly in seahorses, was demonstrated for the first time by Planas et al. [29]. In agreement with those findings, the results achieved in the present study demonstrate the effectiveness of D°_eff_ as temperature-independent developmental index in stable isotopes studies involving different temperature levels. The calculation of D°_eff_ is based on the principle of thermal summation whereby the rate of development is linearly related to environmental temperature above a species-specific threshold temperature (T_o_) at which development is arrested [26]. However, a constrain on the use of D°_eff_ is that T_o_ is unknown for most species and explicit experimental assessments are required for T_o_ estimation at a species level [29].

Low food availability or a delayed initial feeding in seahorse juveniles is accompanied by a progressive decrease in weight and energetic status [38,39]. Newborns deprived of food for 5 days reduced weight, but increased in length at the expense of endogenous reserves consumption. The higher weight loss observed at 21 °C was probably due to both a higher metabolic activity and a lower energetic efficiency compared to juveniles kept at 18 °C and 15 °C. As a consequence, unfed seahorses maintained at 21 °C would consume their body reserves faster than at lower temperatures. Initially, the main catabolic sources would be lipids and, to a lesser extent, proteins [39]. Subsequently, proteins would be almost the unique catabolic source available. In consumers, isotopic discrimination results from the balance between assimilation and excretion processes [40]. In ammonotelic fish, ammonia excretion predominates following hatching as a by-product of an amino acid-based metabolism [41]. There are two components to nitrogenous excretion in fish: endogenous (for maintenance) and exogenous fractions; the former is affected by fish size and temperature levels [42]. Within limits, increasing temperatures accelerate most physiological processes [43], resulting in higher growth rates and reduced excretion rates. A selective decrease in the lighter isotopes δ^12^C (loss of ^12^CO_2_ due to respiration/catabolism) and/or δ^14^N (selective ^15^N-depleted excretion) would be expected in the absence of food [4,16]. Consequently, tissues would become enriched in ^15^N because they are forced to synthetize their own amino acids pool by transamination from tissue proteins. This would result in an inverse relationship between δ^15^N and growth rate, which is also related with the reported increase of δ^15^N in fasting animals [16,44,45,46]. The increase in δ^15^N values occurs due to the preferential use of molecules with only light isotopes for catabolism and body retention of those with heavier isotopes [47]. Those processes agree with the slight initial decrease in δ^15^N observed in unfed juveniles, which was followed by a reduced increase of δ^15^N (protein catabolism) until the end of the starvation period (days 5–7, depending on temperature level).

The effects of starvation in the isotopic composition of a variety of fish are rather variable among species. Small increases in δ^13^C values have been reported in unfed larvae of common carp (*Cyprinus carpio*) [13], whitefish (*Coregonus lavaretus*) [48] and pacu (*Piaractus mesopotamicus*) [49]. Changes in δ^15^N values where not detected in pacu larvae, but fasting significantly affected δ^15^N signatures in Nile tilapia (*Oreochromis niloticus*), with values higher than in fed fish [46]. In red drum (*Sciaenops ocellatus*) larvae, isotopic composition was not related to food deprivation [17]. Among other factors, the amount and quality of yolk available in lecitotrophic fish larvae and parental/maternal inheritance would probably define initial isotopic patterns as pointed out in bluefin tuna *Thunnus thynnus* [50]. Our findings suggest that *H. guttulatus* juveniles can support food deprivation for a certain period, as previously reported in other seahorse species [38], which is inversely related to temperature level. Accordingly, juveniles developing at lower temperatures would be less dependent on food availability during the initial planktonic period, enhancing their survival under adverse food availability conditions. However, food deprivation would lead to physiological alterations in juveniles, with implications in specific gravity and buoyancy [29].

Fed juveniles grew faster at 18 and 21 °C than at 15 °C. Juveniles at 15 °C were very likely incapable to assimilate prey as efficiently as faster-growing individuals held at warmer temperatures. The hypothesis of a higher food assimilation rate in seahorses held at warmer temperatures is supported by the shifting in δ^13^C and δ^15^N values of juveniles towards those in prey. Furthermore, the similarity between the isotope composition in unfed and 5 days old fed juveniles suggest a poor assimilation efficiency at the suboptimal temperature of 15 °C, which is near the threshold temperature (T_o_ = 13.1 °C) for *H. guttulatus* juveniles [29]. Due to the absence of yolk reserves and the rapid adaptation to exogenous feeding, juveniles underwent a rapid initial change towards dietary isotopic values. Isotopic shifting in fed juvenile seahorses clearly differed from unfed individuals, which was very likely due to changes in the ratio of anabolism to catabolism and to metabolic disruptions derived from fasting in the former [29]. δ^13^C and δ^15^N values in unfed juveniles increased and decreased, respectively, but not significantly.

Effects of temperature and feeding on isotopic enrichment have been reported in several species [18,19,30,46,51,52]. In metamorphosed winter flounder *Pseudopleuronectes americanus*, higher lipid content at a lower temperature was responsible for the increase in δ^13^C values [30]. Temperature-dependent nutrient assimilation rates (indicated by stable isotope data) have also been demonstrated for summer flounder *Paralichthys dentatus* [31] and larval red drum *Sciaenops ocellatus* [17]. All those studies were carried out considering development as chronological time. Considering that development scale, the decrease in isotopic rates observed in *H. guttulatus* juveniles until day 5 was directly related with temperature level (Figure 2). Conversely, the effect of temperature level resulted negligible when using D°_eff_ as development progress scale. This finding is probably related to a well-known limitation in early developing *H. guttulatus*, e.g., the low digestion efficiency on the days following first feeding [53,54], particularly when fed on *Artemia* [55]. Such issue would apply to all temperature conditions. Consequently, a reduced effect of temperature would be expected under such conditions as confirmed when referring development as D°_eff_.

From day 5, the progressive decrease in δ^13^C and increase in δ^15^N towards diet isotope values suggests an enhancement in prey digestion/assimilation, particularly from day 15 onwards, which agrees with gut development in the species [20]. About day 15, significant changes occur in gut morphology and physiology, including a change in the secretion of goblet cells and a progressive increase in the intestinal absorption surface. Those changes would lead to better digestive efficiencies and significant enhancement of digestion and assimilation capabilities from that age onwards.

Growth in fish [24,25,56], and specifically in seahorses [27,28,29], is generally linked to variation in temperature but not always [57]. In ectotherms, faster metabolism in δ^15^N and particularly in δ^13^C should theoretically increase at warmer temperatures [19,30,43,58,59], with some exceptions [57,60]. The faster daily growth rates and greater daily isotopic changes occurring in seahorse juveniles at 18 and 21 °C compared to 15 °C is consistent with the cornerstone of metabolic theory [61].

When using D°_eff_, the increased growth in seahorses fed at 21 °C was likely due to an increase in metabolic activities when compared to lower temperatures. Considering growth, nutrient assimilation and survival, the optimal temperature for juvenile seahorse performance would be achieved at temperatures of 18 °C or slightly higher (19–20 °C), which is in accordance with previous findings [29]. The results have a practical applicability to ex-situ rearing techniques of the species, particularly on the optimization of temperature levels. This will contribute to optimize breeding programs for the conservation of the species (wild population’s recovery), an approach that could counteract fishing pressure on threatened stocks [35,62,63]. The use of D°_eff_ also permitted the development of a growth model independent of temperature with the use of a unique equation [29].

Our findings are relevant to some aspects of the biology and ecology of *H. guttulatus*, such as the geographical distribution of the species or the duration and extension of the breeding season. In nature, *H. guttulatus* has adapted to different temperature ranges along its distribution from Morocco to the British Isles [64]. The duration of the breeding season differs on the region considered but extends over the warmer period of the year when primary and secondary production is maximal [65,66]. The results from this study show that water temperature is an important determining factor for growth, food assimilation, and survival of *H. guttulatus* juveniles. Seahorses inhabiting temperate or sub-tropical areas would experience enhanced growth and survival under optimal prey availability compared to those from colder regions [29]. The effect of climate change, with increasing water temperatures within the Atlantic range of distribution for *H. guttulatus*, might affect seahorse physiology and their biogeographical distribution [29]. Considering the current distribution range of the species, increased temperatures would (a) provide a rich food supply, (b) increase potential colonization of coastal areas beyond the current Northern limit of the species, and (c) improve juvenile performance in terms of assimilation and metabolism.

## 5. Conclusions

We provided new insights for the understanding of growth and food assimilation in early developing *Hippocampus guttulatus* juveniles under different temperature levels. One of the main goals of this study was to demonstrate the practical use of D°_eff_ as a developmental scale progress on the assessment of isotopic patterns for the first time. The present study highlights the importance of considering temperature when interpreting stable isotope data, especially in field-collected specimens from populations that consistently experience a fluctuating temperature regime. Further comparative studies on the effects of temperature in developing seahorses are also encouraged, as well as for ground-truthing the applicability of results from mesocosm experiments to field populations.

## Figures and Tables

**Table 1:** Survival, dry weight, standard length (SL) and C:N ratios in fed and unfed *Hippocampus guttulatus* juveniles maintained at 15, 18 and 21 °C. Weight and size change correspond to the difference between the initial value (day 0) and the value of the corresponding sampling day. Data are provided as means (two batches per temperature level) and standard deviations (s.d.). n: individuals sampled. SL: standard length.

Treatment	Temp	Day	D°_eff_	n	Survival	Dry Weight (mg)		Weight Change (mg)		SL (mm)		Size Change (mm)		C:N	
	(°C)				(%)	mean	sd	mean	sd	mean	sd	mean	sd	mean	sd
Onset	15	0	0	10	100	0.80	0.18	−	−	15.30	0.69	−	−	2.80	0.05
Fed	15	5	12.5	4	94	0.86	0.12	0.06	0.18	17.22	0.86	1.92	0.16	2.92	0.11
	15	15	31.5	4	44	1.11	0.39	0.31	0.21	18.08	1.70	2.78	1.01	2.85	0.02
	15	30	60.0	4	22	1.53	0.39	0.73	0.21	21.32	2.98	6.02	2.28	2.83	0.01
	18	5	24.5	4	100	0.81	0.42	0.01	0.24	17.84	0.42	2.53	0.28	2.92	0.06
	18	15	73.5	4	93	2.65	1.63	1.85	1.45	23.92	5.46	8.62	4.76	2.89	0.03
	18	30	147.0	4	86	7.57	7.28	6.77	7.10	30.49	12.98	15.19	12.29	2.92	0.07
	21	5	36.5	4	100	1.32	0.86	0.52	0.69	19.58	4.05	4.28	3.36	2.88	0.11
	21	15	115.5	4	96	4.49	2.45	3.69	2.27	29.37	4.53	14.07	3.83	2.86	0.01
	21	30	234.0	4	81	12.79	10.20	11.99	10.03	39.13	12.15	23.73	11.31	2.82	0.10
Unfed	15	5	12.5	10	88	0.76	0.24	−0.04	0.06	16.34	0.37	1.03	0.33	2.87	0.01
	18	5	24,5	10	94	0.57	0.11	−0.23	0.07	16.35	0.70	1.05	0.00	2.94	0.01
	21	5	36.5	10	89	0.61	0.02	−0.20	0.16	16.16	0.70	0.86	0.01	2.81	0.08
